# The Dissector’s Manual

**Published:** 1884-09

**Authors:** 


**The Dissector's Manual.** By W. BRUCE CLARKE, M. A., M. B., F. R. C. S. Demonstrator of Anatomy and Operative Surgery, St. Bartholomew's Hospital, London, etc., etc., and C. B. LOCKWOOD, F. R. C. S. Illustrated with 49 engavings. Philadelphia: H. C. Lea's Son & Co.

The above are the last four issues of the " Students' Series of Manuals" which the enterprising house of H. C. Lea's Son & Co. are now publishing. These volumes are admirably calculated to meet the growing demands for concise and authoritative manuals on the various branches of medical science in a cheap and portable form. The authors are well-known teachers, who have presented their subjects in concise and clear language. So far as issued we have most heartily to commend the volumes. Not only will they prove of immense value to students, but they will serve the practitioner well, as works for quick reference. The volumes are of convenient size to carry in the pocket, and contain from 400 to 500 pages each. They are accurately and profusely illustrated, and bound in red limp cloth. The other volumes already issued are—

